# An alternative model for (breast) cancer predisposition

**DOI:** 10.1038/s41523-017-0017-7

**Published:** 2017-04-17

**Authors:** Erik Teugels, Sylvia De Brakeleer

**Affiliations:** Molecular Oncology, Faculteit Geneeskunde en Farmacie, Vrije Universiteit Brussel, Laarbeeklaan 103, Brussel, 1090 Belgium

## Abstract

While environmental factors can greatly increase cancer risk, it is clear that an individual’s genetic constitution has strong impact on tumor formation. Hereby we present an alternative cancer predisposition model built on the assumption that efficiencies of DNA maintenance mechanisms in normal cells are similar but not identical for each person. Small variations in an individual’s genetic constitution may result in slightly increased genomic instability and generate typical mutational signatures in normal cells. With recent and expected advances in the next-generation sequencing field, qualitative and quantitative establishment of such mutational signatures in normal tissue must become feasible, and may meanwhile provide a more accurate estimation of individual cancer risks, even in persons without familial antecedents. An additional advantage of this approach is that cancer risk assessment will not strictly rely on the individual’s genetic identity, but will also consider other factors (e.g., environmental and age) that can affect genomic integrity.

## Introduction

Human life starts with a fertilized egg that after zillions of divisions generates an adult person. This developmental process generates a lot of cell types organized in tissues. Although different cell types express different combinations of proteins, (almost) all these cells have an identical genome. Each cell has to follow tightly an epigenetically defined developmental program to ensure the growth and survival of the organism. Cancer can arise when one particular cell succeeds to escape from its pre-programmed developmental path. This cell no longer behaves as a good team player but acquires properties that allow it to escape (induced) death, to start or continue dividing without responding to the inhibitory signals from the host, to escape from immunosurveillance, to organize into tumor masses that succeed to infiltrate nearby healthy tissue (invasion). Some tumor cells are released from the primary tumor mass, reach the circulation, and can eventually regenerate new tumor masses (metastasis), finally leading to the death of the host.

Apparently, the most efficient way for a cell to acquire all these malignant properties is to accumulate somatic mutations in specific genes that support the molecular pathways involved for instance in cell division, apoptosis, and cellular interactions. Such genes are generally called cancer genes that can accumulate driver mutations. Recently, however, a few examples have been described where tumorigenesis appeared to be supported exclusively by an epigenetic mechanism.^1^

The number of somatic mutations in cancer genes needed for a normal cell to acquire a malignant cancer phenotype has been previously estimated between two and eight.^2^ Since mutational events are rather random, a lot of somatic mutations will be without consequences (called passenger mutations), others will be detrimental or lethal to the cell, and only a few will be able to contribute to the cancer phenotype. Since cancer occurs mostly not more than once in a lifetime, it must be very challenging for a cell to accumulate the required number of somatic mutations finally leading to cancer. In the past decades, genetic analyses on tumor samples revealed that a large set of genes can contribute to tumorigenesis.^3^ However, each tumor type is characterized by somatic driver mutations that are often found in the same gene(s). In lung cancer for instance, *EGFR* mutations and *KRAS* mutations are mutually exclusive and found each in about 25% of non-small cell lung cancers.^4^ In melanoma, about half of the cases present a typical mutation in the *BRAF* gene.^5^ In the exocrine pancreas, the large majority of carcinomas contain a *KRAS* mutation.^6^ It seems thus that, for a specific tissue, the number of mutational paths a normal cell can follow to become a tumor cell is rather limited.

In the nineties, investigations in the genetic basis of cancer predisposition made notable progress. Colon cancer and breast cancer (BC) predisposition genes were discovered,^7–10^ and remarkably, the majority of these genes were involved in the molecular processes responsible for the maintenance of DNA integrity. These observations led to the conclusion that a primary requirement for a normal cell to become a cancer cell would be the acquisition of a mutator phenotype responsible for the increased incidence of somatic mutations.^11, 12^

## Why do we get cancer: the classical view based on the BC example

At the time of their discovery, the BC predisposition genes *BRCA1* and *BRCA2* fitted nicely into the tumor suppressor gene (TSG) model designed by Knudson^13^ and Comings.^14^ Indeed, an inherited heterozygous mutation in *BRCA1* ^9^ or *BRCA2* ^10^ (mostly a protein truncating mutation) is responsible for the increased BC risk in the family. In female mutation carriers, the BC risk would rise from 10 to about 80%. In heterozygous condition, such mutation would not harm the normal functions of a cell. Only when the second (wild type) allele is inactivated in a particular cell (e.g., by gene locus deletion or promoter inactivation), this cell would acquire a mutator phenotype that increases its chances to accumulate the required number of somatic mutations in order to become a cancer cell. According to the classical model for cancer predisposition, increased cancer risk would thus result from the fact that one of the few mutations needed to generate a tumor is already present in the germline (the one step ahead concept).

Unfortunately, germline mutations in *BRCA1* and *BRCA2* can explain high cancer risk only in a fraction (~20%) of the BC families. Additional efforts were initiated to identify new BC predisposing genes, leading to the discovery of *CHEK2*.^15^ Also this gene is involved in the maintenance of DNA integrity, and a recurrent protein truncating mutation (c.1100delC) is responsible for increased cancer risk in a fraction of BC families. However, the uncomplicated co-segregation pattern seen in *BRCA1* and *BRCA2* families, where (almost) all BC patients carry the predisposing mutation and where mutation carriers have a high risk to develop cancer (typical for high penetrant monogenic diseases), is not found in *CHEK2* families. The BC risk associated to *CHEK2* mutation carriership only doubles in such families and a significant fraction of BC patients do not carry the *CHEK2* mutation.^15, 16^ Consequently, many authors proposed that BC predisposition in *CHEK2* families occurs according to a polygenic model. Mutations in several BC predisposing genes would segregate in such families and are, together, responsible for the observed high BC risk. Unfortunately, these “other” genes remain still unknown, rendering the genetic counseling of a *CHEK2* family less obvious. It remains also unclear whether these different genes contribute independently to the increased cancer risk or in a combinatory way. Today, BC predisposing properties have been assigned to several more genes like *P53*, *ATM*, *PALB2*, *BRIP1*, *BARD1*, and so on. Although the molecular mechanisms in which these genes are involved are known, the impact of mutations in those genes on BC risk remains often unclear, merely because these mutations were detected in a very small number of families that complicates the establishment of genotype/phenotype correlations. It is commonly accepted now that highly penetrant mutations in new BC predisposing genes can be expected to occur only in a very small subset of BC families and are therefore much more difficult to identify and characterize. In contrast, it has been suggested that the majority of orphan BC families have a polygenic basis.^17^ The discovery of the underlying genes remains cumbersome and a better understanding of their mode of action would surely contribute to their identification.

In a polygenic model, several genes can contribute independently to BC predisposition. Clustering of BC cases on a pedigree would result from the clustering of “moderate penetrant” BC gene alleles in that part of the pedigree. Stratton and Rahman nicely described this situation having the *CHEK2* c.1100delC mutation in mind.^18^ Within a high BC risk family, carriers of a *CHEK2* mutation could acquire a mutator phenotype in a particular cell when loss of heterozygosity occurs at the corresponding wild-type allele. However, since these persons have also a substantial chance to carry a second or even a third moderate penetrant BC gene allele, a mutator phenotype can also be acquired through wild-type allele loss of one of those other genes. Consequently, wild-type *CHEK2* allele loss in the tumor of a c.1100delC mutation carrier would not necessarily mean that the mutator phenotype was acquired through *CHEK2* inactivation, as another BC predisposing gene could have been inactivated before.^18^ This model also predicts that the BC risk for a female c.1100delC mutation carrier is higher when she has first degree relatives with BC as compared with a female carrier with no or more distant relatives with BC. Although attractive by its simplicity, this polygenic model has been challenged more recently (at least concerning *CHEK2*) by the observation that homozygous c.1100delC carriers are viable.^19^

Alternatively, in a polygenic model the different moderate BC predisposing genes might contribute to a mutator phenotype in a combinatory manner. The molecular mechanisms responsible for the maintenance of DNA integrity are often supported by huge protein complexes. Some of these proteins might be essential, their (functional) loss leading inevitably to cell death. Loss of other proteins, like BRCA1, is still compatible with cell growth and survival (at least in some tissue types, e.g., breasts and ovaries, but with implications on DNA stability and consequently tumor generation) but does not allow the development of whole organisms. On the other hand, functional loss of a single moderate BC predisposing gene product might be without consequences for the cell or organism, but a mutator phenotype would arise when the product of a second moderate BC predisposing gene becomes deleterious. According to the TSG-based model, the corresponding wild-type alleles of two different BC predisposing genes of moderate penetrance would need to be inactivated in the same cell in order to acquire a mutator phenotype. This seems unlikely however, since inactivation of one particular wild-type allele (as is expected for *BRCA1* and *BRCA2* mutation carriers) is already a very exceptional event.

More recently, genome-wide association studies (GWAS) have revealed a set of single-nucleotide polymorphisms (SNPs) associated with BC risk in non- selected BC patients^20^ (BC susceptibility factors), but also in sub-cohorts such as *BRCA1* mutation carriers^21^ (and therefore called genetic modifiers of cancer risk). GWAS investigating 211,155 SNPs with a minor allele frequency above 5% could identify 77 BC risk-associated variants. The risks conferred by such common low-risk variants are not at all sufficiently large to be useful in risk prediction individually, but it was proposed that their combined effect might achieve sufficient discrimination power for use in population-based programs of BC prevention and early detection.^22, 23^ Mavaddat et al.^24^ constructed a mathematical tool to calculate the polygenic risk score (PRS) for BC that allowed stratification of BC risk in women with or without a family history of BC. This PRS assumes that the contributing SNPs act independently (PRS = sum of the minor alleles weighted by the per allele log odds ratio (OR)). The 77 BC risk-associated SNPs, their respective OR, and the genes they may be associated with are presented in Supplementary Table 4 of reference 24. Interestingly, 50 of these 77 SNPs could be linked to 42 unique genes, 13 of them being related to mechanisms of DNA synthesis/repair or cell cycle control (according to the Gene Ontology biological processes tool from Ensembl, see also Supplementary Table 1). However, risk discrimination by genomic profiling using GWAS data has still its limitations and cannot be used for individual counseling. While additional common low-risk variants will probably be added to the 77 SNPs already listed, the GWAS approach remains blind for all risk variants (low, intermediate, and high) present at lower incidences in the studied population. Also lifestyle/environmental risk factors are not considered.

Having their increasing number in mind, it is rather difficult to believe that all the cancer predisposing genes detected by GWAS would function according to the TSG-based model. Obviously, the classical model for (breast) cancer predisposition needs refreshment.

## Why do we get cancer: an alternative model

**Fig. 1:**
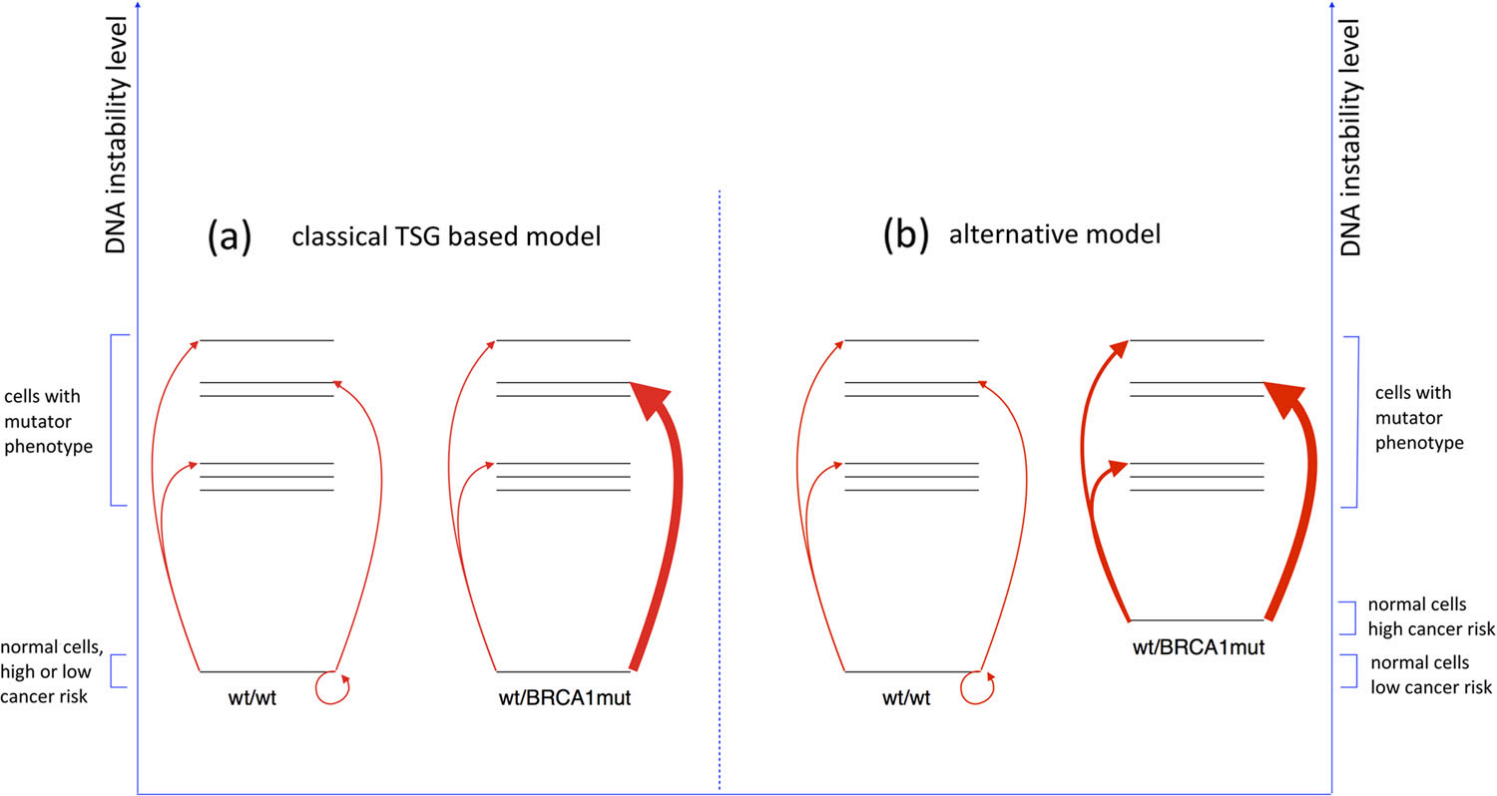
Mechanism leading to the acquisition of increased genomic instability in normal cells from persons with or without a highly penetrant germline mutation conferring enhanced risk for cancer (e.g., a *BRCA1* mutation). A normal cell can acquire enhanced genomic instability (a mutator phenotype, one of the first steps in tumorigenesis) through a one-step (dominant mutations) or a two-step (recessive mutations) mechanism. According to the classical TSG-based model (**a**), normal cells from all individuals present the same genomic stability. Individuals without a cancer predisposing mutation (wt/wt) have a low probability to acquire a mutator phenotype through the accumulation of specific mutations (*red arrows*). Normal cells from a high cancer risk person (wt/BRCA1mut) can acquire increased genomic instability in the same way as wt/wt cells, but preferentially by inactivation of the remaining wild-type *BRCA1* allele (the thickness of the arrows reflects the relative probability at which the event will occur). In the alternative model (**b**), normal cells from a person with a cancer predisposing germline mutation would already manifest a slightly increased genomic instability resulting in the faster accumulation of somatic mutations. This increased genomic instability would be the primary cause for a cancer predisposed cell to acquire a mutator phenotype. In cells with a highly penetrant germline mutation, the mutator phenotype is preferentially acquired through inactivation of the remaining wild-type allele. Without the increased genomic instability associated to this germline mutation in normal cells, this “second hit” will most probably not occur

It is widely accepted that (breast) cancer predisposing mutations (e.g., in *BRCA1*) are recessive, and that all (normal) cells from a heterozygous mutation carrier are phenotypically identical to the cells of a non-carrier. However, several authors showed that leukocytes and fibroblasts from *BRCA1* and *BRCA2* mutation carriers accumulate significantly more DNA damages when submitted in vitro to non-physiological stress situations.^25–27^ A significant decrease in the number of BRCA1 containing nuclear foci in untreated leukocytes freshly collected from *BRCA1* and *BRCA2* mutation carriers has also been observed.^28^ A recent study investigating the functionality of BRCA1 in the heterozygous state reported that several functions (e.g., double-strand break repair by homologous recombination) are well executed, while other functions (e.g., stalled replication fork repair) suffer from BRCA1 haploinsufficiency.^29^ Taken together, these observations suggest that the multi-protein complexes responsible for DNA synthesis/repair/maintenance perform somewhat less efficiently in cells with a heterozygous *BRCA1* or *BRCA2* mutation under physiological conditions, and that this slight increase in DNA instability might be the primary cause for the increased BC risk in mutation carriers. *BRCA1* and *BRCA2* mutation carriers would thus have a slightly increased probability to acquire a mutator phenotype in a particular cell preferentially by inactivation of the remaining wild-type *BRCA1* or *BRCA2* allele, although other strategies are not excluded (Fig. 1). In turn, the cells that got the mutator phenotype meanwhile acquired enhanced capacities to become cancer cells through the increased incidence of mutations in gatekeeper^12^ genes. Although this adaptation to the classical TSG-based model for cancer predisposition might appear rather semantic at first, this revised model is, however, able to propose a mechanistic explanation for genetic cancer predisposition when several genes are involved.

**Fig. 2:**
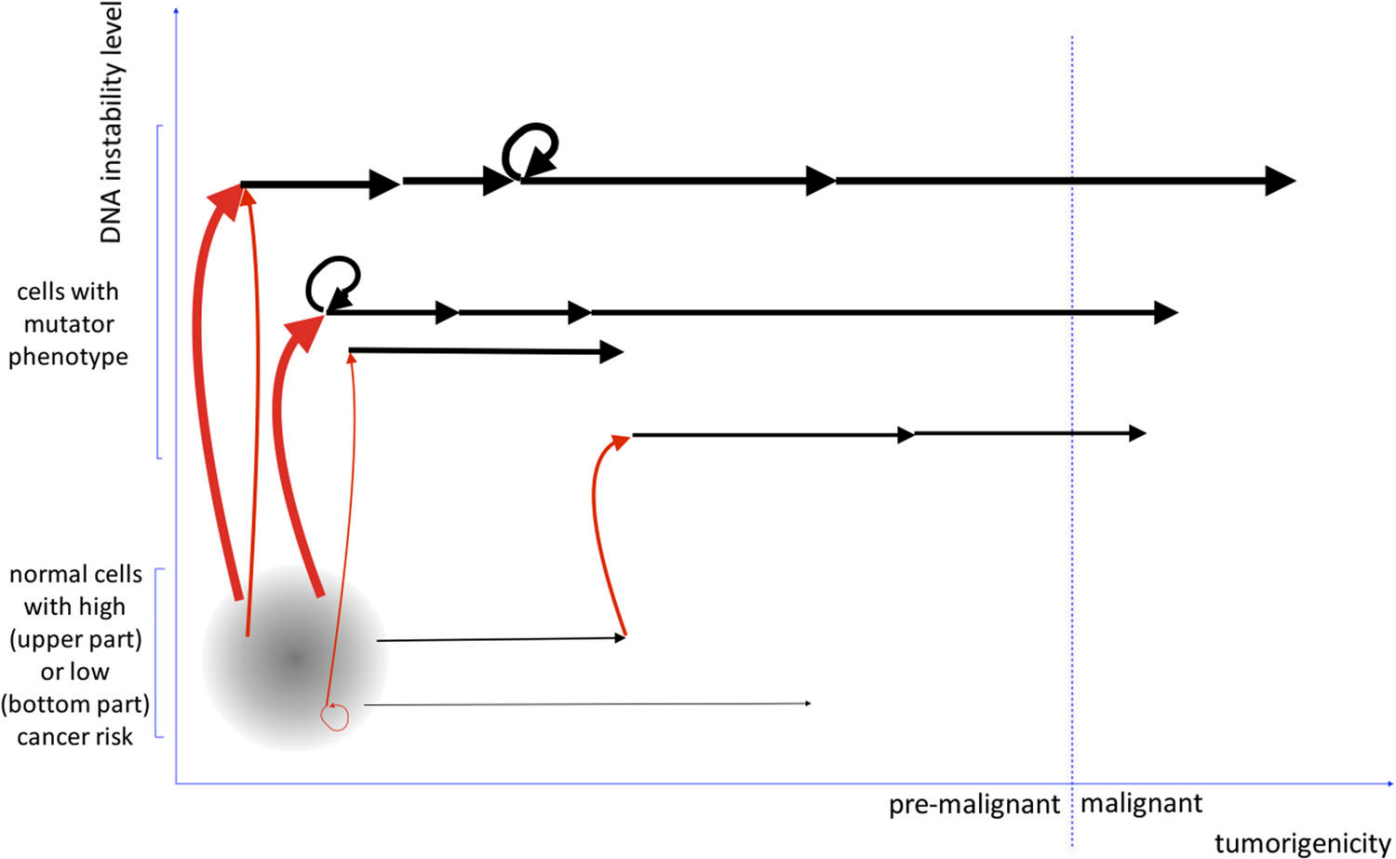
Genetic mechanism leading to the acquisition of a malignant cancer phenotype in normal cells from a particular tissue. A normal cell from a specific individual has an intrinsic DNA instability level and can be represented as a dot located within the *gray area* on the figure. The higher the cellular instability, the higher the cell is represented on the Y-axis. The position on the Y-axis for a particular individual is defined by the combined genotype of all his genes involved in DNA surveillance (caretaker genes). Mutational events occur more frequently in cells with higher DNA instability, and can generate a mutator phenotype when affecting caretaker genes (*red arrows*). By analogy, cellular characteristics involved in tumorigenicity such as division rates, apoptosis, cellular interactions, … can jointly be plotted on the X-axis as regards to their contribution to the cancer phenotype of the cell. Mutations (*black arrows*) occurring in the genes supporting these underlying molecular mechanisms (gatekeeper genes) will arise more frequently in cells with higher genomic instability. Normal cells can already manifest an inherent higher risk to acquire a cancer phenotype due to the fact that they bear particular germline variants in their gatekeeper genes (normal cells from such individuals are plotted in the most right part of the *gray area*). It might be expected that many cells engaged in the tumorigenic process will not have the time to reach the malignant phenotype or will be eliminated before. Similar figures can be plotted for the different tissue types

BRCA1 and BRCA2 function within huge molecular complexes^30^ responsible for maintenance of DNA integrity, complexes made up of many different protein types. Many more genes are probably coding for these proteins than the 20–40 genes investigated with the actually available NGS-based commercial kits designed for the detection of cancer predisposing mutations. These genes often contain a lot of polymorphic variants, some variants being more prevalent in the population than others. The cells from two different persons will thus contain DNA surveillance complexes that are built up with slightly differing proteins, and even within a same cell these protein complexes may differ due to genome diploidy. Although these DNA surveillance complexes will all perform in a very similar way, it is rather difficult to admit from an evolutionary perspective that they will all function with identical efficiencies. Some combinations of polymorphic variants might be slightly detrimental, other rather neutral or even beneficial. Some genes such as *BRCA1*, *BRCA2*, and *CHEK2* may carry a protein truncating mutation known to elicit nonsense-mediated decay (NMD) of the corresponding mRNA. Since the efficiency of this mutant RNA degrading mechanism is not necessarily absolute,^31^ one might expect that heterozygous truncating *BRCA1* and *BRCA2* mutations can impair the DNA surveillance complexes either by haploinsufficiency or by the presence of truncated protein forms. At which extend a cancer predisposing protein truncating mutation (e.g., in *BRCA1* or *BRCA2*) will impair DNA surveillance complexes can also depend on the presence of particular polymorphic forms in other proteins participating in the same complexes (called modifier genes in this case). The efficiency of the DNA surveillance mechanism may thus differ slightly from person to person, but also from tissue to tissue: the efficiency of the NMD mechanism can be cell-type dependent and promoters regulating the expression of the proteins participating in the DNA surveillance complexes can be differentially activated according to cell type. Cells with lower DNA surveillance capacities will have a higher probability to accumulate somatic mutations and consequently to acquire a mutator phenotype than cells with higher DNA surveillance capacities, although other factors such as mitotic rates^32^ and cellular environment will concomitantly influence the speed at which somatic mutations accumulate (Fig. 2). According to the proposed model, typical mutational signatures^33^ would be expected in normal cells and quantitative assessment of these signatures might allow discrimination of persons at risk for cancer, at least when investigating the tissue at risk. It is known since decades that normal cells can accumulate specific mutations when submitted in vitro to UV irradiation (cytosine to thymidine substitutions or C > T). A same mutational signature (C > T) was later on detected in skin carcinoma.^34^ Lung cancers from smokers however are overwhelmed by C > A changes, a mutational signature generated by tobacco carcinogens.^35^ Since the emergence of the NGS technology, a large number of cancer genomes could be sequenced, each one containing thousands of somatic mutations (a few driver mutations but mostly passenger mutations). These mutations are scars of perturbated biologic processes involved in DNA synthesis and repair, each process generating a characteristic mutational signature.^36, 37^ These signatures can involve single nucleotide substitutions but also more complex alterations. Using the catalog of somatic mutations from thousands of cancers and applying appropriated mathematical models, it became possible to extract the different mutational signatures present in a single tumor sample.^33^ At least 18 different mutational signatures contribute to the catalog of somatic mutations in BC, and specific rearrangement signatures were found in cancers with inactivating *BRCA1* or *BRCA2* mutations.^38^ Since all cancers are clonal cell populations generated from single normal cells, the set of somatic mutations obtained from a tumor sample with the actual NGS technologies correspond to the mutations present in the progenitor cell of the final dominant clonal expansion of the analyzed sample. In principle, these mutational signatures can be generated before or after the initiation of the cancer process. Recently, it was shown that two mutational signatures must have been generated in the normal tumor precursor cells since the mutation loads for these two signatures are positively correlated with the age at diagnosis of the cancer.^39^ This study included 1170 BCs and confirmed that the biological processes generating the two mutational signatures are active in the normal precursor cells. However, the determination of specific mutational signatures directly in normal tissue is much more challenging as normal tissue samples can be considered polyclonal (the different cells constituting the collected tissue sample containing frequently different somatic mutations) and also the mutation rates in these cells are expected to be significantly lower. However, by generating clonal expansions of adult stem cells from three different tissue types (small intestine, colon, and liver) and analyzing their respective content of somatic mutations, researchers succeeded to confirm the presence of tissue-specific mutational signatures in normal cells.^40^ When a normal cell acquires a mutator phenotype (after a mutational hit in a caretaker gene), a different mutational signature will most probably be generated that is much more easily identified in the resulting tumor tissue. Very interesting in this respect, a mutational signature already present before acquisition of the mutator phenotype associated to DNA mismatch repair deficiency, and distinct from the mutational signature associated to this mutator phenotype, has been described in microsatellite instability cancers.^41^ Indeed, the type of mutator phenotype that has been acquired by a cancer cell may depend on the type of germline variations present in the genes encoding the DNA surveillance proteins. Even more recently, Alexandrov et al.^39^ provided data in strong support of our model. They investigated a large set of cancer genomes (including BC genomes) and could identify two different mutational signatures with underlying mutational processes that, according to the authors, must already be active in normal cells.

Individuals with the highest genomic instability in their normal cells will have the highest probability to get cancer. When this genomic instability is conferred mainly by a single genetic variant such as a *BRCA1* mutation, pedigree analysis will reveal a clear autosomal dominant transmission for cancer predisposition, but not all female carriers will develop the disease as penetrance is not absolute. Determination of genetic instability via mutational signatures in normal cells might thus enable discrimination between high-risk and lower-risk *BRCA1* mutation carriers. On the other hand, when this genomic instability results from the co-occurrence of several genetic variants (polygenic), familial clustering of the disease will often be much less obvious. Many sporadic cancer cases probably belong to this last category, the patients being genetically predisposed (with a genetic instability detectable in their normal cells) while the hereditability of the disease is hidden.

Currently, genetically based cancer risk estimations in single individuals rely almost solely on mutation carriership in single high penetrant cancer predisposition genes. Unfortunately, only a very small fraction of persons can benefit from this approach. The ultimate aim will be reached when all genes involved in cancer predisposition will be determined, all pathogenic variants in these genes identified and their contribution to cancer predisposition evaluated in each possible combination for each tissue. While this sounds as distant future, a new approach is suggested by the hereby proposed model (meanwhile allowing validation of the model). Each cell would have an intrinsic DNA instability level that can differ from cell type to cell type and from person to person. The higher the DNA instability level of a cell, the higher the risk that this cell will generate a tumor. On the other hand, recent and expected advances in the next-generation sequencing technology (longer reads, single molecule sequencing, lower error rates, and so on) will allow the assignment of a mutational signature defined qualitatively and quantitatively to each normal tissue type (possibly after in vitro clonal enrichment). One may expect that this mutational signature will reflect cancer risks, at least for the investigated tissue. Qualitative assessment of the mutational signatures might be indicative for the underlying germline gene alterations and may facilitate their identification, but might also help to identify a possible contribution of external factors. Quantitative assessment will in turn help to quantify the cancer risk. The recently developed CRISPR/CAS technology allows the generation of subclones harboring a single heterozygous mutation. In a cell line derived from a normal cell (no mutator phenotype) of a person considered with low cancer risk after pedigree analysis, it is possible now to introduce a heterozygous mutation in a cancer risk-associated gene (e.g., *BRCA1*) and to compare the mutational signatures obtained before and after in vitro mutagenesis. We expect that specific mutational signatures will be associated with specific gene defects and their characterization will help in the interpretation of mutational signatures observed in vivo. The analysis of mutational signatures obtained in vitro can help to determine the effects generated by single and combined variants in genes associated to cancer risk, as well as their mutual interaction with external (mutagenic) factors.

Since the accumulation of mutational signatures in normal cells is a lifetime process, the quantitative assessment of specific mutational signatures will generate time-dependent outcomes correlating with the cancer risk at tissue sampling. When comparing a group of individuals, the relative scores obtained for their cancer risk-associated mutational signatures will thus depend on the genomic constitution of each of these persons, their lifetime exposure to internal or external mutation inducing agents, the investigated tissue type, as well as their age.

Finally, one might also expect that other molecular mechanisms than those contributing to genomic instability modulate cancer risk. Indeed, two different tissue types from a same person may present similar genomic instability scores suggesting similar mutation rates (also in caretakers and gatekeeper genes), but their potency to evolve toward malignancy may strongly diverge due to, for instance, differential efficiencies in the clearing of cells that initiated tumorigenesis. Therefore, investigating mutational signatures in easily accessible tissues such as blood may still be relevant for the evaluation of cancer risk in other tissues.

## Conclusion

In the classical TSG-based model for cancer predisposition, the presence of one particular heterozygous mutation in the constitutive DNA of a person will result in the shortening of a mutational path that normal cells can follow in order to become a cancer cell. Increased cancer risk for a specific patient would thus exclusively result from the possibility for his normal cells to follow that shortened mutational path. With this model, cancer risk estimations can only be performed when the causal genes and the variants they harbor are well characterized, which is seldom the case.

According to the alternative model presented hereby, increased cancer risk would primarily result from a slightly increased genomic instability already present in the normal cells of a person. This increased genomic instability can be the consequence of the particular genetic constitution of that person and can also result from exposure to internal or external mutagenic factors. Interestingly, this genomic instability could be monitored qualitatively and quantitatively by investigating mutational signatures in normal cells, thanks to recent or expected advances in NGS-based technologies. While qualitative data would provide information about the genes responsible for increased cancer risk and also reveal the contribution of external factors, quantitative data would in turn allow a better estimation of cancer risk.

In real-life situation we do not exclude that global cancer risk can result from both the shortening of a mutational path (especially when the cancer predisposing mutations are not located in caretaker genes) and the pre-existing genomic instability in normal cells. However, we are convinced that in the majority of cancer cases pre-existing genomic instability is the major component.

## Electronic supplementary material

Supplementary Table 1
